# Notch signaling facilitates hepatitis B virus covalently closed circular DNA transcription via cAMP response element-binding protein with E3 ubiquitin ligase-modulation

**DOI:** 10.1038/s41598-018-38139-5

**Published:** 2019-02-07

**Authors:** Zijing Wang, Kazunori Kawaguchi, Masao Honda, Shinichi Hashimoto, Takayoshi Shirasaki, Hikari Okada, Noriaki Orita, Tetsuro Shimakami, Taro Yamashita, Yoshio Sakai, Eishiro Mizukoshi, Seishi Murakami, Shuichi Kaneko

**Affiliations:** Department of Gastroenterology, Kanazawa University Graduate School of Medical Science, Kanazawa, Japan

## Abstract

Notch1 is regulated by E3 ubiquitin ligases, with proteasomal degradation of the Notch intracellular domain affecting the transcription of target genes. cAMP response element-binding protein (CREB) mediates the transcription of hepatitis B virus (HBV) covalently closed circular DNA (cccDNA). We assessed the relationship between HBV cccDNA and Notch signaling activities. HBV cccDNA levels and relative gene expression were evaluated in HBV-replicating cells treated with Jagged1 shRNA and a γ-secretase inhibitor. The effects of these factors in surgically resected clinical samples were also assessed. Notch inhibition suppressed HBV cccDNA and CREB-related expression but increased ITCH and NUMB levels. Proteasome inhibitor augmented HBV cccDNA, restored Notch and CREB expression, and inhibited ITCH and NUMB function. Increased HBV cccDNA was observed after ITCH and NUMB blockage, even after treatment with the adenylate cyclase activator forskolin; protein kinase A (PKA) inhibitor had the opposite effect. Notch activation and E3 ligase inactivation were observed in HBV-positive cells in clinical liver tissue. Collectively, these findings reveal that Notch signaling activity facilitates HBV cccDNA transcription via CREB to trigger the downstream PKA-phospho-CREB cascade and is regulated by E3 ubiquitin ligase-modulation of the Notch intracellular domain.

## Introduction

Hepatitis B virus (HBV) infection affects more than an estimated 400 million people worldwide, increasing their risk of liver cirrhosis and hepatocellular carcinoma^1^. HBV covalently closed circular DNA (cccDNA), which is assembled into histone-containing viral minichromosomes, serves as a template for the transcription of viral mRNA and is regulated by preC/C, S1, S2, and X promoters. The persistence of HBV cccDNA is the major obstacle to the elimination of chronic HBV infection and this DNA is insensitive to antiviral drugs^2^, enabling viral rebound and drug resistance upon antiviral treatment discontinuation.

Notch signaling is a highly conserved intercellular signaling pathway that is crucial to various aspects of liver function, including development, repair and regeneration, inflammation, and hepatocarcinogenesis^3–5^. The basic molecular elements in this signaling pathway include two types of ligands (Jagged [Jag-1/-2)] and Delta-like [Dll-1/-3/-4]), four Notch receptors (Notch-1/-2/-3/-4), and various transcription factors. Notch signaling is initiated by the binding of ligands to its corresponding receptors followed by release of the intracellular domain of the receptor (NICD) by two proteolytic cleavages (α/γ secretase) and subsequent translocation of the NICD to the nucleus to modulate downstream gene expression.

E3 ubiquitin ligase plays an important role in Notch receptor regulation. ITCH, an E3 ubiquitin ligase that belongs to the HECT family, negatively regulates Notch1 signaling by specifically activating its ubiquitination and promoting ubiquitination-dependent proteasomal degradation of the NICD. Furthermore, NUMB can interact with ITCH to cooperatively enhance Notch ubiquitination and degradation, circumventing its nuclear localization and downstream activation of Notch1 target genes^6,7^.

Various transcription factors have been linked to HBV, such as cAMP response element-binding protein (CREB), which mediates HBV transcription by binding to the cAMP response elements located on the preS2*/*S or X promoter of the HBV viral genome^8,9^. The transcriptional coactivator CREB-binding protein (CBP) is also recruited to cccDNA to support HBV transcription through acetylation of cccDNA-bound H3 histones and transcription factors^10^. In addition, Notch can bind to CREB to exert an effect on CREB-dependent gene transcription and induce CREB phosphorylation^11,12^. However, the relation between Notch signaling and CREB/CBP in HBV cccDNA regulation remains unclear.

Recent studies showed that HBV X protein activates Notch signaling in HBV-related HCC^13,14^. Notch is abundantly expressed in chronic hepatitis B patients^15^. As HBV replication is a major cause of HBV-related HCC, we hypothesized that Notch signaling may perform an important regulatory role not only in HBV-related HCC, but also in HBV replication. To test this hypothesis, we examined the changes in HBV DNA, cccDNA, HBV RNA, E3 ubiquitin ligases, and Notch and CREB pathway-related gene expression, as well as their relations, in HBV-replicating cells by inhibiting or activating Notch signaling. We also immunohistochemically assessed these factors in surgically resected clinical samples.

## Results

### Notch inhibition suppresses HBV cccDNA

**Figure 1:**
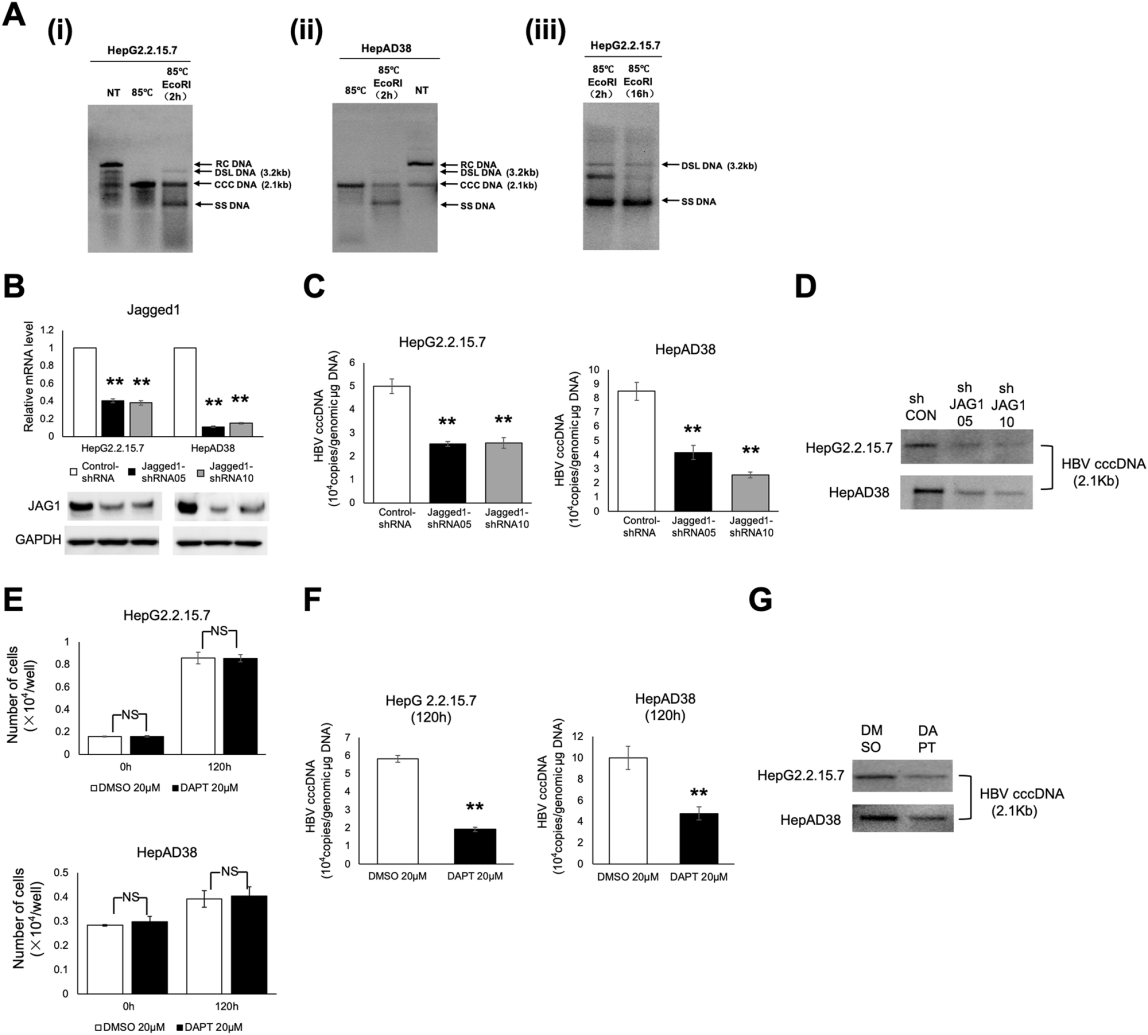
Notch inhibition markedly suppresses hepatis B virus (HBV) covalently closed circular DNA (cccDNA). (**A**) HBV Hirt DNA was extracted from HepG2.2.15.7 (i) and HepAD38 (ii) cells and detected by Southern blotting under the condition of no treatment, 85 °C for 5 min, or 85 °C for 5 min plus EcoRI 2 h digestion. (iii) EcoRI digestion of HepG2.2.15.7 cells for 2 h and 16 h after 85 °C treatment. (**B**) Jagged1 mRNA and protein levels were significantly decreased after transfection with Jagged1 shRNA, as detected by RTD-PCR (upper) and western blotting (below). (**C**) RTD-PCR quantification of cccDNA accumulation in HepG2.2.15.7 and HepAD38 cells transfected with lentiviruses encoding Jagged1 short hairpin RNA (shRNA). (**D**) Southern blot analysis confirming decreased cccDNA levels in HepG2.2.15.7 and HepAD38 cells transfected with Jagged1 shRNA. (**E**) No significant change in cell numbers at 0 and 120 h between 20 µM DMSO and DAPT treatment. (**F**) RTD-PCR quantification of cccDNA accumulation in HepG2.2.15.7 and HepAD38 cells administered 20 µM DAPT. (**G**) Southern blot analysis confirming decreased cccDNA levels in HepG2.2.15.7 and HepAD38 cells treated with 20 µM DAPT. cccDNA results are expressed as the number of cccDNA copies per genomic μg (mean ± standard deviation) from three independent experiments. *P < 0.05 and **P < 0.01 versus corresponding control shRNA values or 20 µM DMSO. Abbreviations: NT, no treatment; RC DNA, relaxed circular DNA; DSL DNA, double-stranded linear DNA; SS DNA, single-stranded DNA; shCON; Control-shRNA, shJAG1; Jagged1 shRNA; NS, not significant.

HBV cccDNA in Hirt extracts of HBV-replicating cells was analyzed by Southern blot. The heat-resistant cccDNA band in HBV-replicating cells migrated to the 2.1-kb position under no treatment conditions or after being heated to 85 °C. Consistently, after EcoRI digestion and linearization, the cccDNA band shifted to a 3.2 kb double-stranded linear (DSL) position (Fig. 1A,(i,ii)). Due to incomplete digestion of EcoRI, a cccDNA band could still be detected after 2 h EcoRI treatment. However, the cccDNA band was completely linearized after 16 h of EcoRI digestion (Fig. 1A,(iii)). The original gel image with size marker is provided in Fig. S1.

To identify the involvement of the Notch signaling pathway in HBV cccDNA amplification, we silenced this pathway with several shRNAs targeting the Notch ligand Jagged1, and found Jagged1 silencing by shRNA-05 and -10 markedly inhibited Jagged1 expression and the HBV cccDNA level (Figs 1B–D and S2). We also used the γ-secretase inhibitor DAPT, which restricts cleavage of the Notch receptor and disturbs NICD signal transduction^16^. Although several reports showed an inhibition of HepG2 cell growth after DAPT treatment^17^, we did not observe any significant change in the growth of HBV-replicating cells after 120 h DAPT treatment under our experiment conditions (Fig. 1E) and suppose that the distinct characteristics between hepatoma and HBV-replicating cells due to HBV transfection may account for the discrepancy in cell viability after DAPT treatment^18^. Interestingly, there was clear attenuation of the HBV cccDNA levels after DAPT treatment (Fig. 1F,G). These data show that inhibition of Notch signaling decreased HBV cccDNA levels. We also found similar inhibition of HBV DNA, HBV total RNA, HBsAg, and HBeAg after Notch suppression (Fig. S3), suggesting that Notch signaling contributed to HBV facilitation. To figure out whether other ligands were also involved in Notch signaling-mediated HBV facilitation, we generated several siRNAs for silencing other Notch ligands, including Jagged2, DLL1, and DLL4, and checked the cccDNA level, but did not find a similar decrease after transfection with their siRNAs (Fig. S4).

### E3 ubiquitin ligase regulates Notch signaling and HBV cccDNA levels

**Figure 2:**
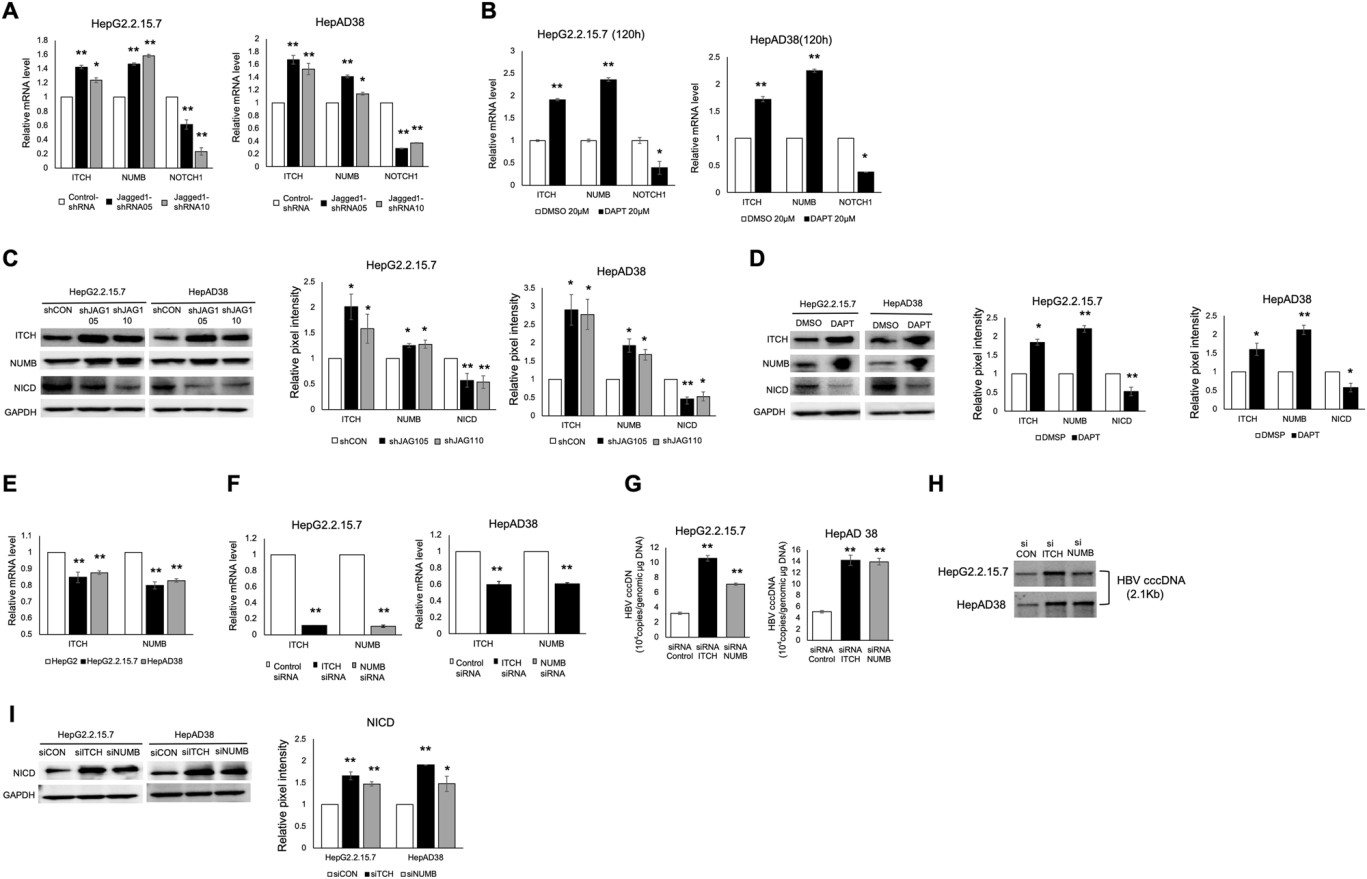
E3 ubiquitin ligases regulate Notch intracellular domain and intranuclear HBV DNA levels. (**A**) Levels of E3 ubiquitin ligase genes (ITCH and NUMB) were upregulated and NOTCH1 was downregulated in HepG2.2.15.7 and HepAD38 cells transfected with Jagged1 shRNA. (**B**) E3 ubiquitin ligase gene expression was upregulated and Notch1 expression was downregulated in HepG2.2.15.7 and HepAD38 cells after 20 µM DAPT treatment for 120 h. (**C**) Western blotting confirmed the protein level trends of the E3 ubiquitin ligases and Notch-related markers in Jagged1 shRNA-transfected cells. Relative pixel intensities (right) were normalized to the GAPDH pixel intensity (Control-shRNA = 1). (**D**) Western blotting revealed the same protein level trends of the E3 ubiquitin ligases and Notch-related markers after 20 µM DAPT treatment. Relative pixel intensities (right) were normalized to the GAPDH pixel intensity (DMSO = 1). (**E**) Gene expression levels of ITCH and NUMB were decreased in HBV-replicating cell lines. (**F**) Gene expression levels of ITCH and NUMB were significantly inhibited by ITCH and NUMB-siRNA. Blocked expression of ITCH and NUMB by siRNA significantly increased the HBV cccDNA level, as determined by RTD-PCR (**G**) and Southern blotting (**H**). (**I**) NICD protein expression was increased after siITCH and siNUMB transfection. Relative pixel intensities (right) were normalized to the GAPDH pixel intensity (Control-siRNA = 1). Quantitative gene expression data represent the mean ± standard deviation of three independent experiments and were normalized to the expression levels of human GAPDH. *P < 0.05 and **P < 0.01 versus the corresponding control shRNA and DMSO (Control-shRNA, DMSO = 1). Abbreviations: shCON, Control-shRNA; shJAG1, Jagged1 shRNA; siCON, Control-siRNA; siITCH, ITCH-siRNA; siNUMB, NUMB-siRNA.

E3 ubiquitin ligases ITCH and cooperator NUMB negatively regulate Notch1 by promoting NICD ubiquitination and degradation^6,7^. We thus determined whether ITCH and NUMB were involved in the Notch signaling pathway and Notch-mediated upregulation of HBV cccDNA. Notch inhibition by Jagged1 shRNA and DAPT suppressed Notch1 and NICD, indicating diminished Notch pathway activity, and was accompanied by elevated levels of ITCH and NUMB mRNA and protein (Fig. 2A–D), suggesting that the E3 ubiquitin ligase ITCH and its cooperator NUMB could negatively modulate the Notch signaling pathway. Next, we detected lower ITCH and NUMB mRNA expression in HBV-replicating cells compared with HepG2 cells (Fig. 2E), suggesting that E3 ligase activity was inhibited in HBV-replicating cells. To identify whether ITCH and NUMB were involved in Notch-mediated upregulation of HBV cccDNA, we used two specific siRNAs for significantly reducing ITCH and NUMB mRNA expression (Fig. 2F). Decreases in ITCH and NUMB by siRNA increased HBV cccDNA and NICD levels (Fig. 2G–I). Together, this evidence indicates that E3 ubiquitin ligase is essential for restricting Notch-mediated HBV cccDNA facilitation.

### Proteasome inhibitor and NICD overexpression promotes Jagged1/Notch1-dependent signal transduction and augments HBV cccDNA

**Figure 3:**
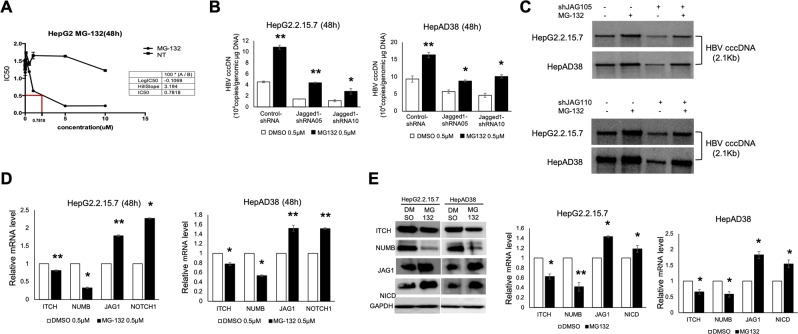
Proteasome inhibitor (MG-132) promotes Jagged1/Notch1-dependent signal transduction and intrahepatic HBV cccDNA accumulation. (**A**) IC50 of MG-132 after 48 h treatment of HepG2 cells. (**B**) RTD-PCR analysis of the cccDNA level in HepG2.2.15.7 and HepAD38 cells transfected with Jagged1 shRNA with or without MG-132 (0.5 µM) treatment for 48 h. Results are expressed as the number of cccDNA copies per genomic μg (mean ± standard deviation) from three independent experiments. *P < 0.05 and **P < 0.01 versus the corresponding 0.5 µM DMSO. (**C**) Southern blot analysis confirming upregulated cccDNA levels in control and Jagged1 shRNA-transfected HepG2.2.15.7 and HepAD38 cells after treatment with 0.5 µM MG-132 for 48 h compared with DMSO. (**D**) E3 ubiquitin ligases were downregulated and Notch-related genes were upregulated after MG-132 administration. (**E**) Western blot analysis of the levels of E3 ubiquitin ligases and Notch-related markers after MG-132 administration. Relative pixel intensities (right) were normalized to the GAPDH pixel intensity (DMSO = 1). Quantitative gene expression data represent the mean ± standard deviation of three independent experiments and were normalized to the expression levels of human GAPDH. *P < 0.05 and **P < 0.01 versus the corresponding control shRNA and DMSO (Control-shRNA, DMSO = 1). Abbreviations: shCON, Control-shRNA; shJAG1, Jagged1 shRNA.

Because Notch1 can be degraded by the proteasome through a ubiquitin-dependent mechanism, we next assessed whether disruption of this degradation would affect Notch1-dependent intrahepatic HBV replication. The proteasome inhibitor (MG-132) was used after calculation of the IC50 (Fig. 3A). Interestingly, increased HBV DNA, HBV cccDNA, HBV total RNA, HBsAg, and HBeAg levels in both control and Jagged1 shRNA-transfected cells were observed after 48 h administration of MG-132, suggesting that proteasome degradation inhibition not only promoted intrahepatic HBV replication, but also abolished Jagged1 shRNA-mediated HBV inhibition (Figs 3B,C and S5). Furthermore, MG-132 administration increased the mRNA and protein levels of Jagged1, Notch1, and NICD and decreased ITCH and NUMB levels in HBV-replicating cells (Fig. 3D,E). Hence, inhibition of ubiquitin-dependent proteasomal degradation further elevated Notch signaling-mediated cccDNA activation.

Notch has a proline-, serine-, and threonine-rich (PEST) sequence at its C-terminus, which is known to be involved in protein degradation^19^. We next investigated whether overexpression of Notch signaling or removal of the PEST region affected HBV replication. pCMV-XL4-NICD-HA and pCMV-XL4-NICD ΔPEST-HA plasmids were transfected into HBV-replicating cells and successfully activated Notch1 signaling by upregulating the mRNA expression of Notch1 and the Notch1 downstream gene Hes1 (Fig. S6A,B). We next checked the cccDNA expression and found that both NICD and NICD-ΔPEST overexpression upregulated the cccDNA level and abolished Jagged1 shRNA and DAPT-mediated HBV inhibition (Fig. S6C–F), suggesting that activation of Notch signaling facilitated HBV cccDNA transcription. However, deletion of the PEST sequence in NICD did not further elevate the cccDNA level compared with NICD transfection (Fig. S6C–F), which may be due to PEST-independent ubiquitination of Notch by ITCH/NUMB^6,7^.

### A Notch-CREB (pSer133CREB)-CBP cascade mediates HBV transcription

**Figure 4:**
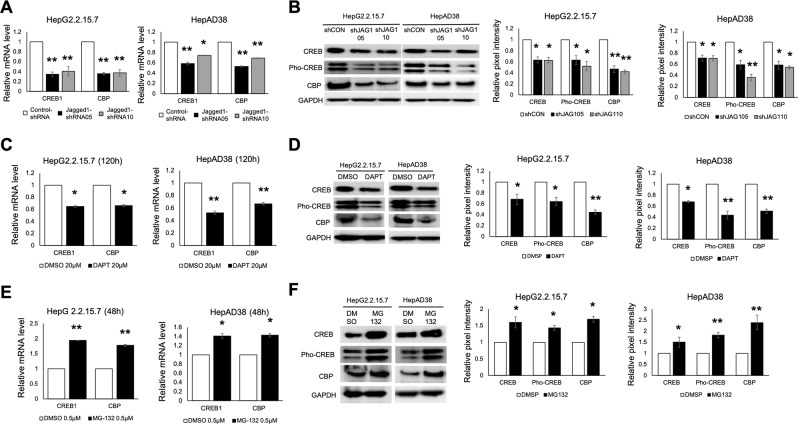
Notch signaling affects cAMP response element-binding protein (CREB) 1 and CREB-binding protein (CBP) gene expression. (**A**) RTD-PCR analysis of CREB1 and CBP gene levels after Notch inhibition by Jagged1 shRNA transfection. (**B**) Protein expression levels were confirmed by western blotting using anti-CREB, anti-phospho-CREB (Ser133), and anti-CBP antibodies after Jagged1 shRNA transfection. Relative pixel intensities (right) were normalized to the GAPDH pixel intensity (Control-shRNA = 1). (**C**) CREB1 and CBP gene expression levels after DAPT treatment. (**D**) Protein expression of CREB, phospho-CREB (Ser133), and CBP after DAPT treatment. Relative pixel intensities (right) were normalized to the GAPDH pixel intensity (DMSO = 1). (**E**) RTD-PCR analysis of CREB1 and CBP gene expression levels after MG-132 treatment. (**F**) CREB, phospho-CREB (Ser133), and CBP protein expression by western blotting after MG-132 treatment. Relative pixel intensities (right) were normalized to the GAPDH pixel intensity (DMSO = 1). Quantitative gene expression data represent the mean ± standard deviation of three independent experiments and were normalized to the expression levels of human GAPDH. *P < 0.05 and **P < 0.01 versus the corresponding control shRNA and DMSO (Control-shRNA, DMSO = 1).

HBV transcription is mediated by transcription factors and coactivators recruited to HBV cccDNA, including CREB and CBP. We next asked whether the transcription factor CREB or its coactivator CBP plays a role in the activation of HBV replication by Notch signaling. RTD-PCR and western blot analysis revealed lower levels of CREB and CBP in Jagged1 shRNA-transfected or DAPT-treated cells (Fig. 4A–D). However, NICD overexpression resulted in CREB and CBP upregulation (Fig. S6G,H).

Because CREB is activated by phosphorylation at Ser133 and Notch induces hyperphosphorylation of CREB^12^, we next studied the phospho-CREB (Ser133) protein level, finding a similar decrease in Jagged1 shRNA-transfected and DAPT-treated cells (Fig. 4B,D). However, MG-132-induced Notch signaling transduction significantly increased CREB, CBP, and phospho-CREB (Ser133) expression (Fig. 4E,F).

**Figure 5:**
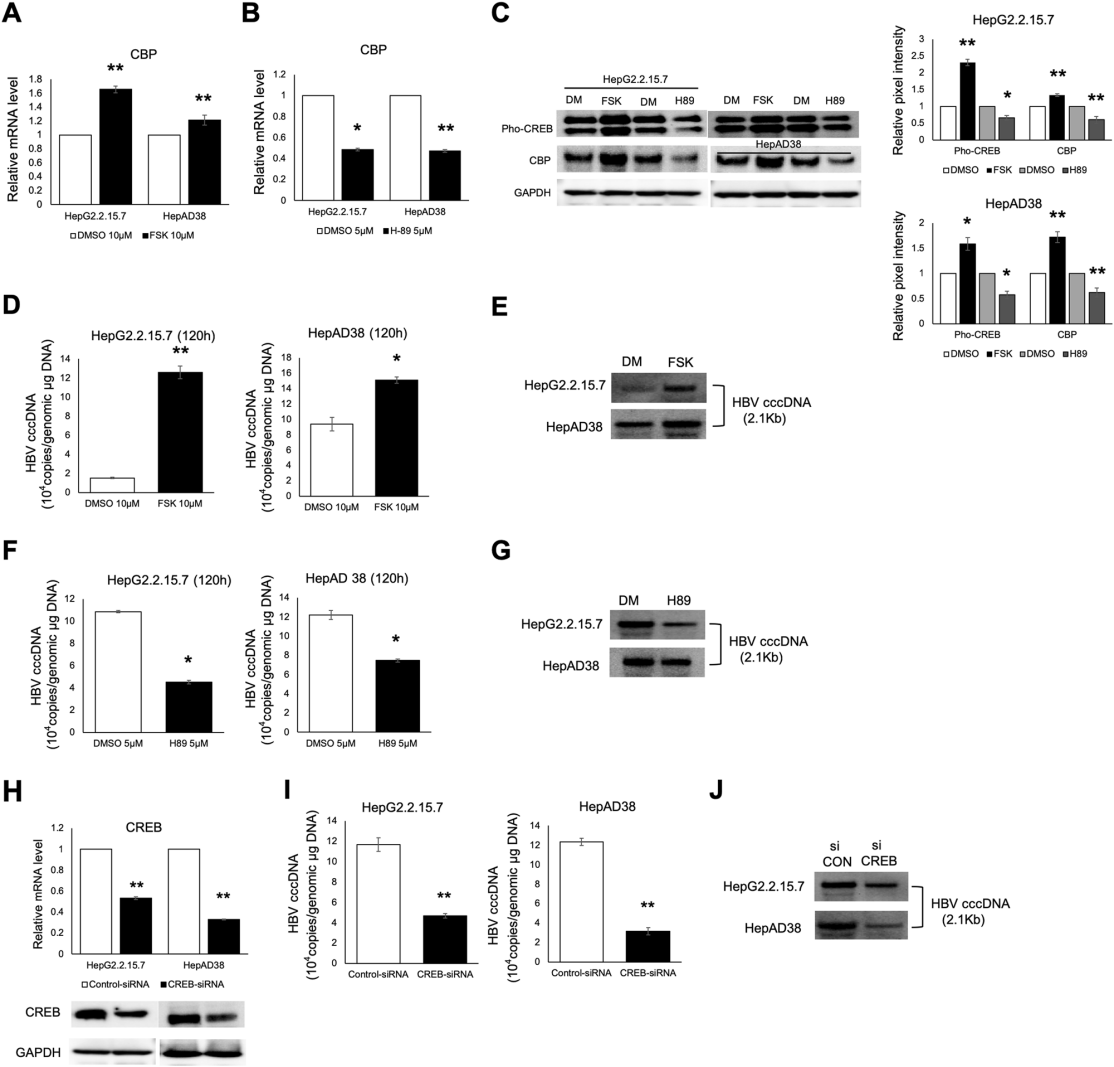
The CREB (pSer133CREB)-CBP cascade mediates HBV replication. RTD-PCR analysis of CBP gene expression after 10 µM Fsk (**A**) and 5 µM H-89 (**B**) treatment for 120 h. (**C**) Western blot analysis for CBP and phospho-CREB (Ser133) protein expression after 10 µM Fsk and 5 µM H-89 treatment for 120 h. Relative pixel intensities (right) were normalized to the GAPDH pixel intensity (DMSO = 1). The HBV cccDNA level was increased in the presence of 10 µM Fsk (120 h), as determined by RTD-PCR (**D**) and Southern blot analysis (**E**). The HBV cccDNA level was decreased in the presence of 5 µM H-89 (120 h), as determined by RTD-PCR (**F**) and Southern blot analysis (**G**). (**H**) CREB mRNA and protein levels detected by RTD-PCR and western blotting were decreased after transfection with CREB-siRNA. (**I**) RTD-PCR quantification of cccDNA accumulation in HepG2.2.15.7 and HepAD38 cells transfected with CREB-siRNA or control-siRNA. (**J**) Southern blot analysis confirming lower cccDNA levels in HepG2.2.15.7 and HepAD38 cells transfected with CREB-siRNA compared with control-siRNA. Results are expressed as the number of cccDNA copies per genomic μg (mean ± standard deviation) from three independent experiments. *P < 0.05 and **P < 0.01 versus the corresponding DMSO. Quantitative gene expression data represent the mean ± standard deviation of three independent experiments and were normalized to the expression levels of human GAPDH. *P < 0.05 and **P < 0.01 versus Control-siRNA (Control-siRNA = 1). Abbreviations: DM, DMSO; siCON, Control-siRNA; siCREB, CREB-siRNA.

To further determine the intrahepatic role of the CREB/CBP pathway, two specific drugs were used. Forskolin (Fsk), an adenylate cyclase activator that induces CREB phosphorylation, increased the expression of phospho-CREB and CBP (Fig. 5A,C) and increased HBV cccDNA copies in both HepG2.2.15.7 and HepAD38 cells (Fig. 5D,E). However, H-89, a potent selective inhibitor of protein kinase A (PKA), had the opposite effect on phospho-CREB (Ser133) and CBP expression (Fig. 5B,C) and HBV cccDNA level (Fig. 5F,G). Additionally, lower expression of CREB by CREB-siRNA also decreased HBV cccDNA copies in HepG2.2.15.7 and HepAD38 cells (Fig. 5H–J). In accordance with Tang *et al*.’s research^20^, CREB inhibition by H89 was shown to inhibit HBV DNA, RNA, HBsAg, and HBeAg, with an opposite effect observed after CREB activation by Fsk (Fig. S7). Collectively, these findings strongly support roles for the Notch-CREB (pSer133CREB)-CBP cascade in HBV intrahepatic replication and demonstrate the possible intranuclear mechanism by which Notch mediates HBV replication through the CREB-CBP signaling axis.

### HBV infection is related to the Notch-CREB-CBP circuit and E3 pathway in clinical HBV patients

**Figure 6:**
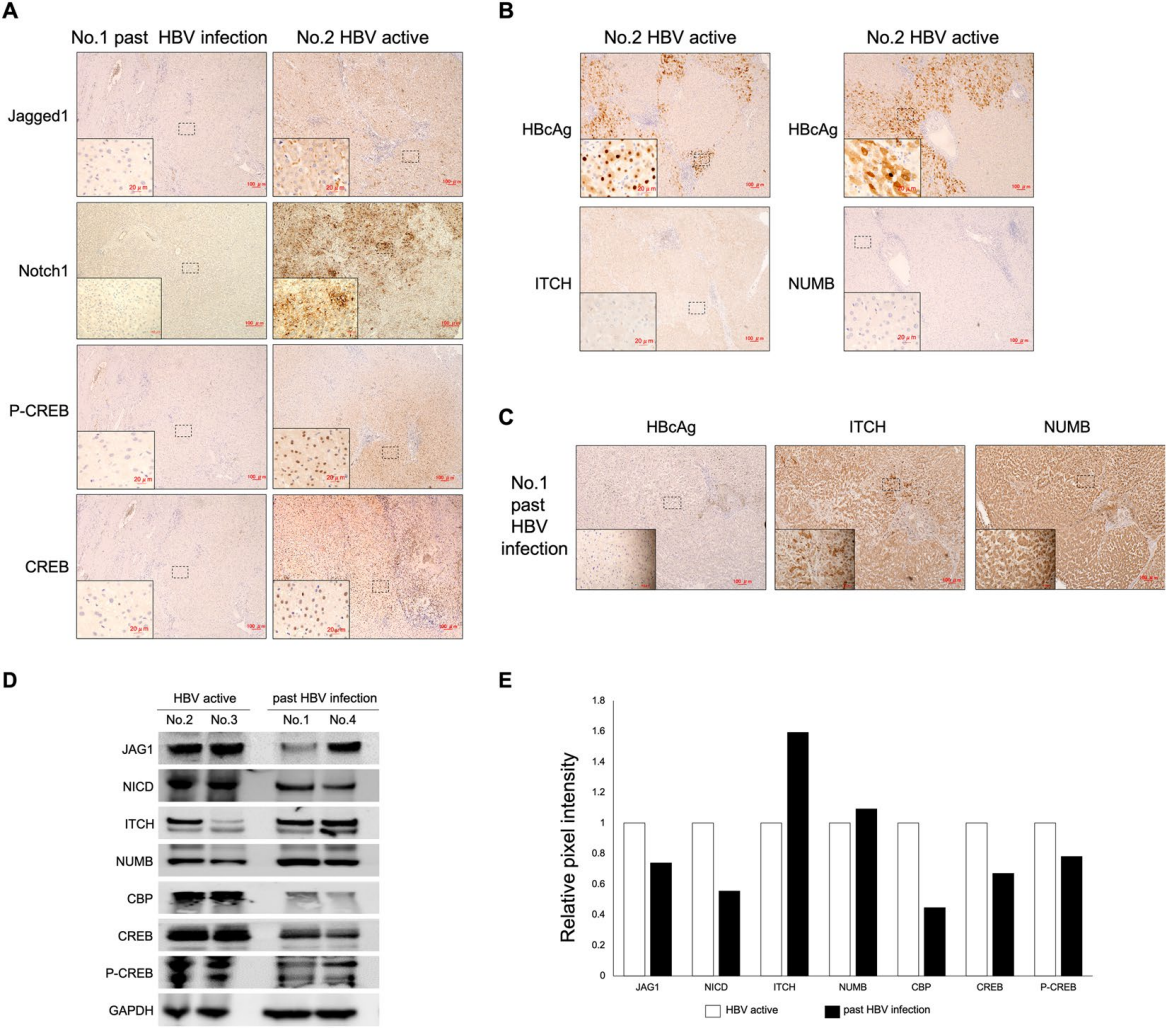
Notch and E3-related expression and the relationship with HBcAg staining in HBV-related clinical liver tissues. (**A**) Immunostaining for Jagged1, Notch1, CREB, and phospho-CREB (Ser133) in liver samples from a patient with past HBV infection and a patient with active HBV. (**B**) Strong cytoplasmic and nuclear staining for HBcAg and negative staining for ITCH and NUMB in the same area of a sample from a patient with active HBV. (**C**) Lack of cytoplasmic and nuclear staining for HBcAg and strong ITCH and NUMB staining in the same area of a sample from a past HBV-infected patient. Original magnification, x200 and x40 (lower left). (**D**) Western blot analysis of protein expression in patients with active HBV and past HBV infection. (**E**) Graphical representation of the mean relative pixel intensity of two patients normalized to the GAPDH pixel intensity (HBV active = 1).

Immunohistochemical and western blot analysis were performed to directly evaluate the effect of HBV status and nucleoside analog treatment on the Notch-CREB-CBP circuit and E3 pathway in HBV-related clinical liver tissues (Table 1). Immunohistochemical staining indicated that the Jagged1-Notch1-CREB-CBP signal was markedly activated in a patient with active HBV (HBsAg, HBeAg, and HBV DNA positive) but inactivated in a patient with past HBV infection (HBsAg, HBeAg and HBV DNA negative) (Fig. 6A). We also compared Notch signaling expression between cells infected with and without HBV and found an upregulation of Notch signaling in HBV-infected cells (Fig. S8). As cytoplasmic or nuclear localization of HBcAg is correlated with active viral replication and greater activity in chronic hepatitis^21^, we investigated whether E3-related expression was correlated with HBV status reflected by HBcAg staining. Interestingly, the HBcAg-stained nuclear and cytoplasmic area in the active HBV sample was negative for ITCH and NUMB (Fig. 6B). However, the HBcAg-negative area in past infection tissues showed strong ITCH and NUMB accumulation (Fig. 6C), suggesting that the E3 pathway was negatively correlated with HBV infection and Notch activity. Western blot results also revealed higher Notch-CREB-CBP and lower ITCH and NUMB expression in patients with active HBV infection compared with past HBV-infected patients (Fig. 6D,E). Taken together, these clinical results from HBV patients further confirmed the increased Notch-CREB-CBP activity under active HBV conditions and the negative role of the E3 pathway in HBV replication.

**Table 1:** Clinical information of HBV-related patients.

Patients No. and HBV infection status	No. 1 (past HBV infection)	No. 2 (HBV active)	No. 3 HBV (HBV active)	No. 4 (past HBV infection)
Age	72	38	52	75
Sex	male	female	male	female
Platelets counts (x10^4^/µl)	20.7	9.5	24.6	18.0
AST (U/L)	16	35	24	17
ALT (U/L)	11	28	19	19
AFP (ng/ml)	<10	1864	29830	<10
Tumor size (mm)	45	50	72	38
Tumor morphology	mod-por	mod-por	mod-por	necrotic nodule*
HBsAg	negative	positive	positive	negative
HBsAb	negative	negative	negative	negative
HBeAg	negative	positive	negative	negative
HBeAb	positive	negative	positive	positive
HBcAb	positive	positive	positive	positive
serum HBV-DNA (Log IU/ml)	<1.3	>8.2	5.4	<1.3
HBV genotype	n/a	C	C	n/a

Abbreviations: mod, moderately differentiated; por, poorly differentiated; n/a, not applicable.

*Post transcatheter arterial chemoembolization (TACE) state.

## Discussion

Notch signaling is an evolutionarily conserved cell interaction mechanism that controls cell fate decisions, proliferation, and apoptosis during developmental and reparative morphogenesis in various organs^22^. Many studies have examined the role of Notch in liver regeneration and repair, as well as in liver metabolism, inflammation, cancer, and HBV infection^3,15,23^. However, much uncertainty remains regarding the mechanism by which the activated Notch pathway contributes to intrahepatic HBV replication and cccDNA amplification. Here, we shed light on the mechanisms underlying Notch-dependent function in HBV regulation. Our results show that HBV replication, reflected by HBV DNA, HBV cccDNA, HBV RNA, and HBV-related antigen, in HBV-replicating cells was markedly inhibited after Jagged1-Notch1 inhibition. Notch signaling is induced by the interaction of Notch ligands (Jagged1 and -2 and Dll1, -3, and -4) with one of the four Notch receptors (Notch1-4). The HBx-Dll4-Notch1 axis has been reported to regulate HBV-mediated HCC^24^. Furthermore, Jagged2 was found to be expressed at a higher level in HCC patients^25^. We ruled out the involvement of other Notch ligands in Notch-mediated HBV replication, as reflected by the maintained level of cccDNA after the silencing of these ligands by the corresponding siRNAs.

Notch signaling inhibition decreased HBV cccDNA levels. Therefore, deregulation of this pathway may break the major obstacle of eliminating chronic HBV infection, relieving viral rebound and drug resistance. Protein ubiquitination is a post-translational modification that is implicated in various cellular processes such as cell cycle progression, signal transduction, protein degradation, endocytic trafficking, and transcriptional regulation. E3 ubiquitin ligases are considered critical for the ubiquitin system by allowing it to selectively recognize, bind, and recruit target proteins for ubiquitination^26^. Several distinct classes of E3 ubiquitin ligases appear to directly regulate the activities of both Notch receptors and their ligands. For example, E3 ubiquitin ligases of the RING family member Mindbomb 1 ubiquitinate the Notch ligand Delta to trigger their activation. However, another RING family, the E3 ubiquitin ligase neuralized, acts negatively on Notch signaling by promoting Jagged1 degradation^27,28^. SEL-10, an F-box protein of the Cdc4 family, may function as a negative regulator of the Notch signaling pathway by targeting NICD degradation^29^.

Unfortunately, we could not detect any evidence that these E3 ligases (Neuralized1, Mindbomb 1, and SEL-10) interacted with Notch signaling and intrahepatic HBV replication (Fig. S9). We considered another E3 ubiquitin ligase from the HECT family, ITCH, because it ubiquitinates Notch and subsequently promotes its degradation by the 26 S proteasome *in vitro* and *in vivo*^7,30^. ITCH deficiency results in defective ubiquitination of Notch1, and thus in augmented Notch1 signaling. Conversely, decreased full-length and cleaved Notch1 are found in ITCH-abundant mice^31^. Furthermore, the vertebrate homolog of Drosophila NUMB also exhibits a regulatory role in Notch signaling by cooperatively enhancing ITCH-catalyzed Notch1 ubiquitination and NICD degradation. NUMB silencing and overexpression also affect the amount of Notch1 and NICD^6^. These studies motivated us to choose ITCH and NUMB for further investigation of Notch regulation.

Surprisingly, we found a negative correlation between Notch signaling and ITCH and NUMB under HBV infection conditions, implying that the increased NICD expression may be due to decreased ITCH and NUMB targeting of NICD for proteasomal degradation. Our work also revealed increased ITCH and NUMB levels after Notch pathway blockage. Although the precise mechanism remains unknown, several studies also indicated an inverse relationship between NUMB and Notch, with a lack of Notch activating NUMB function and Notch inhibition by GSI treatment elevating the NUMB level, which is correlated with the stabilization of the cell fate switch by an asymmetric cell division^32,33^. Inhibition of Notch signaling suppresses the induction of T cell anergy, which might result in upregulation of E3 ubiquitin ligases associated with anergy in lymphocytes, such as c-Cbl, Cbl-b, GRAIL, ITCH, and Nedd4^34,35^. Notch inhibition by GSI also upregulates the ubiquitin-conjugating enzyme E2^36^, which may account for the elevation of E2-E3 complex binding and E3 intrinsic enzymatic activity and contribute to E3 upregulation^37^.

Several studies suggest a role for the ubiquitin/proteasome degradation pathway in controlling Notch signaling^6,7^. MG-132 treatment increased reactive Notch1 species and Notch1 ICD protein levels, suggesting that ubiquitinated Notch is targeted for degradation by the proteasome pathway^29,38^. Based on this research, we suppose that disruption of this degradation by proteasome inhibitor may affect Notch1-dependent intrahepatic HBV replication. Similarly, our work indicated that the ubiquitin/proteasome pathway could indeed modulate Notch signaling transduction, as well as the HBV cccDNA level. The C-terminus of Notch contains a PEST sequence that is implicated in protein degradation. However, the PEST sequence is not required for ITCH/NUMB-mediated Notch ubiquitination. Deletion of the PEST region cannot affect the ubiquitination of NICD by ITCH/NUMB and was unable to further upregulate HBV cccDNA compared with NICD overexpression.

HBV transcription increases binding of CREB to the CREs located at the preS2 region of the HBV genome, which are sensitive to PKA-CREB signaling^9,20^. Both replication and gene expression of HBV require functional CREB and are necessary for the HBV life cycle^8^. Overexpression of CREB mutants inhibits HBV replication and protein production, while inactive CREB abrogates augmentation of cccDNA formation in HepG2.2.15 cells^8,20^. CBP, which acts as a transcriptional coactivator, is also recruited to cccDNA to acetylate cccDNA-bound H3 histones and support HBV transcription^10,39^. Histone hypoacetylation of H3 by CBP is known to promote cccDNA amplification^10^. Notch can bind to CREB to exert an effect on CREB-dependent gene transcription or induce CREB phosphorylation to regulate CREB functions; loss of Notch1 also results in diminished CREB signaling in memory formation^11,12,40^.

Given this evidence, we suppose that the activated Notch signaling in HBV infection may also be linked to the activation of the CREB-CBP cascade to promote HBV transcription. In this study, we defined the role of Notch in activating CREB and the downstream PKA-phosphor CREB cascade, which is fundamental for CREB/CBP-dependent transcription. According to previous research, CREB-CBP may affect the cccDNA level by activating the HBV promoter and changing cccDNA stability via epigenetic modification. The change in the HBV cccDNA level upon CREB and phospho-CREB suppression and activation in our study was also noteworthy. It raises one interesting possibility that, after binding to the HBV promoter, this CREB/CBP complex might stabilize HBV cccDNA through a post-translational modification such as CBP acetylation. Although there is still no direct evidence to prove this hypothesis, the state of HBV cccDNA chromatin does indeed change to a more relaxed form that facilitates transcription after epigenetic modification^41^ and that somehow stabilizes HBV cccDNA and promotes HBV replication. Moreover, reinfection and the intracellular recycling pathway would also contribute to cccDNA amplification^10^.

Here, we propose a regulation loop between HBV intrahepatic replication and Notch signaling transduction (Fig. S10). Initially, cell-to-cell communication from Jagged1 to the Notch receptor initiates Notch signaling and subsequently recruits the CREB-CBP cascade, which facilitates CREB phosphorylation via PKA and then recruits CBP to together translocate to HBV promoter regions of the genome to enhance HBV transcription. After Notch inhibition, ITCH and NUMB were increased and further ubiquitinate NICD to trigger ubiquitination-dependent proteasomal degradation, which may contribute to diminished Notch-CREB signaling transduction and HBV cccDNA facilitation. Overall, although further validation of the *in vivo* relevance of these *in vitro* findings would be worthwhile, the positive feedback regulation loop between HBV intrahepatic replication and the Notch-CREB-CBP cascade activation described here provides new mechanistic evidence that Notch signaling facilitates HBV intrahepatic modulation and offers another therapeutic approach to prevent HBV replication and, hopefully, promote cccDNA clearance.

In conclusion, our data demonstrate that the Notch signaling pathway plays a crucial role in HBV cccDNA facilitation by activating the CREB/CBP cascade. In turn, this triggers activation of HBV transcription, with blockage of this pathway possibly leading to marked inhibition of HBV cccDNA via upregulation of the E3 ubiquitin ligases ITCH-NUMB in a ubiquitin-dependent proteasome-mediated manner.

## Materials and Methods

### Cell culture

HepG2.2.15.7 cells, subcloned from HepG2.2.15 cells, produce a higher titer of HBV than HepG2.2.15 cells^42^. HepAD38, a HepG2-derived cell line, has a stable integration of the entire genome of HBV under tetracycline control^43^. These cell lines were cultured in DMEM/F12 medium (Life Technologies, Carlsbad, CA) supplemented with 10% fetal bovine serum (Sigma, St. Louis, MO), 100 U/mL penicillin, 100 µg/mL streptomycin, 400 µg/mL G418, 10 mM HEPES buffer solution, and 5 µg/mL insulin in a 5% CO_2_ incubator at 37 °C. Cells were harvested at the indicated time points. Before these cell lines could be used, (i) the Gene Recombination Experiments Committee in Kanazawa University approved the experiments, including any relevant details; and (ii) we confirmed that all experiments were performed in accordance with relevant guidelines and regulations.

### Hirt DNA extraction, Southern blot analysis, and real-time detection PCR (RTD-PCR) quantification of HBV cccDNA

The Hirt protein-free DNA extraction procedure was used to isolate HBV cccDNA from HBV-infected cells^44^. HBV preS/S fragments were obtained by PCR amplification with the appropriate forward (5′-TTTTGAATTCATGGGAGGTTGGTCTTCCAAACC-3′) and reverse (5′-TTTTGCGGCCGCTCAAATGTATACCCAAAGACAAAAGA-3′) primers (TaqMan, Thermo Fisher Scientific, Waltham, MA). The amplified HBV preS/S fragments were inserted into a pSPT18 vector to generate a pSPT18-pres/s plasmid. The pSPT18-pres/s template was linearized by HindIII (Takara, Shiga, Japan) and *in vitro* transcription was performed with 1 µg linearized DNA template using a DIG RNA Labeling Kit (Roche, Basel, Switzerland) in the presence of T7 RNA polymerase to generate digoxigenin-UTP-labeled, single-stranded RNA probes. Hirt-extracted DNA was electrophoresed on 1.2% agarose gels and blotted onto a Hybond^TM^-N + membrane (GE Healthcare, Amersham, UK). DIG-labeled single-stranded RNA probes transcribed from pSPT18-pres/s plasmids were used to detect HBV cccDNA. Hybridization was performed with a 30-min pre-hybridization at 50 °C in 10 mL of DIG Easy Hyb™ buffer (Roche) and with overnight hybridization at 50 °C in 3.5 mL of pre-warmed DIG Easy Hyb™ buffer containing 1 µg of DIG-labeled probe (Roche). The membranes were then washed with a Wash and Block Buffer Set (Roche) according to the manufacturer’s instructions. Probe-target hybrids were localized with 2 µL of anti-digoxigenin-AP conjugate (Roche) and detected by 1 mL CSPD (Roche). Images were acquired with the ChemiDoc™ Touch Imaging System (Bio-Rad, Hercules, CA). Hirt-extracted DNA was digested as previously reported^45^. HBV cccDNA expression was quantified with TaqMan Gene Expression Master Mix (Thermo Fisher Scientific) using a specific HBV cccDNA probe (5′-FAM-ACCACCGTGAACGCCCA-MGB-3′), forward primer (5′-GCGCACCTCTCTTTACGCG-3′), and reverse primer (5′-GCCCCAAAGCCACCCA AG-3′) (FASMAC, Kanagawa, Japan) as follows: 50 °C for 2 min, 95 °C for 10 min, and 45 cycles of 95 °C for 10 min and 65 °C for 30 s. HBV DNA and cccDNA copy number was quantified relative to a known copy number of HBV DNA.

### RNA extraction and RTD-PCR analysis

Cells were collected at the indicated time points, and total RNA was isolated by a High Pure RNA Isolation Kit (Roche). A High-Capacity cDNA Reverse Transcription Kit (Thermo Fisher Scientific) was used for first-strand cDNA synthesis with 0.1 μg of total RNA from each sample. RTD-PCRs were conducted using TaqMan Gene Expression Assay Identification according to the manufacturer’s instructions. Quantitative gene expression data were normalized to the expression levels of the housekeeping gene GAPDH (Thermo Fisher Scientific).

### Western blot analysis

Protein extracts were prepared with RIPA Lysis Buffer (Merck Millipore, Temecula, CA) containing Protease Inhibitor Cocktail and phosphatase inhibitor tablets (Sigma). Cells or liver tissues were lysed on ice for 15 min. After ultrasonic extraction, the lysate was centrifuged at 15,000 rpm for 10 min at 4 °C. Protein content was determined using a protein assay kit (Bio-Rad). Proteins (20–40 µg) were subjected to SDS-PAGE electrophoresis and then transferred to Immobilon™-P PVDF Transfer membrane (Merck Millipore) and blocked with 5% skim milk for 1 h at room temperature. Membranes were incubated with primary antibodies at 1:500-1:1000 overnight at 4 °C. Antibodies were purchased from Abcam. After three 5-min TBST washes, the membranes were incubated with the appropriate secondary antibodies (Cell Signaling Technology, Danvers, MA) for 1 h at room temperature. After three TBST washes, signals were visualized with an ECL detection system (Bio-Rad) and ChemiDoc™ Touch Imaging System (Bio-Rad).

### shRNA and siRNA transfection of Notch ligand and transcription factor

HEK293T cells were co-transfected with lentiviral vectors carrying short hairpin RNA (shRNA) targeting the Notch ligand Jagged1 (target sequences are listed in Supplementary Table 1), and the resulting supernatant was collected after 48 and 72 h. Scrambled shRNA was used to control for the non-specific effects of the transfected shRNAs. Transduction was performed by mixing lentivirus aliquots with standard medium containing 10 µg/mL puromycin to remove untransfected cells. Gene-specific endoribonuclease-prepared small interfering RNA (siRNA; Sigma) for human ITCH and NUMB and SignalSilence^®^ CREB-siRNA II (Cell Signaling Technology*)* were used to silence gene expression. For siRNA transfection, cells were seeded 1 day before transfection without antibiotics in the culture medium and grown to 50% confluency on a 6-well plate. Transfection using Lipofectamine 2000 was performed following the manufacturer’s protocol. RNA interference efficiency was determined by RTD-PCR.

### Notch, proteasome, protein kinase A inhibition, and adenylate cyclase activation drug treatment

Cultured cells were administered a Notch signaling-specific blocker, γ-secretase inhibitor (GSI) N-[N-(3, 5-difluorophenacetyl)-l-alanyl]-S-phenylglycine t-butyl ester (DAPT), at 20 µM (Peptide Institute, Osaka, Japan) for 0, 24, 48, 72, 96, and 120 h. DAPT-containing medium was replaced at 24, 48, 72, and 96 h. Total RNA, DNA, and protein were isolated at 120 h. Cell viability was analyzed at 0 and 120 h on a microplate reader at 450 nm. Before proteasome inhibitor (MG-132) (Peptide Institute) treatment, the IC50 of MG-132 after 48 h administration for HepG2 cells was calculated and then the cells were treated with 0.5 µM MG-132 for 48 h. RNA and DNA were isolated at the indicated time points. The adenylate cyclase activator forskolin (Fsk; Sigma) and protein kinase A (PKA) inhibitor H-89 (Sigma) were used at 10 and 5 µM, respectively. Medium was changed at 24, 48, 72, and 96 h. Total RNA, DNA, and protein were isolated at 120 h.

### Immunohistochemical staining of patients’ clinical samples

We immunohistochemically stained surgical samples from HBV-related hepatocellular carcinoma patients to determine whether HBV infection was related to Notch signaling activity in terms of the ubiquitin-proteasome system. All study participants provided informed consent, and the study design was approved by an ethics review board. This study was approved by the medical ethics committee of Kanazawa University to use stored clinical liver tissue samples (approval number, 346). Formalin-fixed, paraffin-embedded background samples of the liver were analyzed according to HBV infection status (Table 1). Slides were incubated with primary (Abcam, London, UK) and secondary (Dako, Santa Clara, CA) antibodies. Western blot analysis was also performed to analyze related protein expression levels in these clinical samples. All of these methods were performed in accordance with the relevant guidelines and regulations.

### Statistical analysis

All pairwise data are presented as mean ± standard deviation and were analyzed by Student *t* test using SPSS 22.0 (SPSS, Chicago, Illinois). *P* values of < 0.05 were considered significant. IC50 values were determined by GraphPad Prism 5 software (GraphPad Software, La Jolla, CA). Western blot intensity was calculated using ImageJ v1.8 software (ImageJ, Bethesda, MD).

## Supplementary information

Supplementary Info
