# Supplementary material for: Notch signaling facilitates hepatitis B virus covalently closed circular DNA transcription via cAMP response element-binding protein with E3 ubiquitin ligase-modulation
Source: Sci Rep. 2019 Feb 7;9:1621. doi: 10.1038/s41598-018-38139-5 (PMC6367350; doi:10.1038/s41598-018-38139-5)
Supplement: Supplementary file 1 — Supplementary Info [file 41598_2018_38139_MOESM1_ESM.docx]

**Notch signaling facilitates hepatitis B virus covalently closed circular DNA transcription via cAMP response element-binding protein with E3 ubiquitin ligase-modulation**

Zijing Wang^1^, Kazunori Kawaguchi^1^*, Masao Honda^1^, Shinichi Hashimoto^1^, Takayoshi Shirasaki^1^, Hikari Okada^1^, Noriaki Orita^1^, Tetsuro Shimakami^1^, Taro Yamashita^1^, Yoshio Sakai^1^, Eishiro Mizukoshi^1^, Seishi Murakami^1^ & Shuichi Kaneko^1^

^1^Department of Gastroenterology, Kanazawa University Graduate School of Medical Science, Kanazawa, Japan

**Supplementary Figures**

**
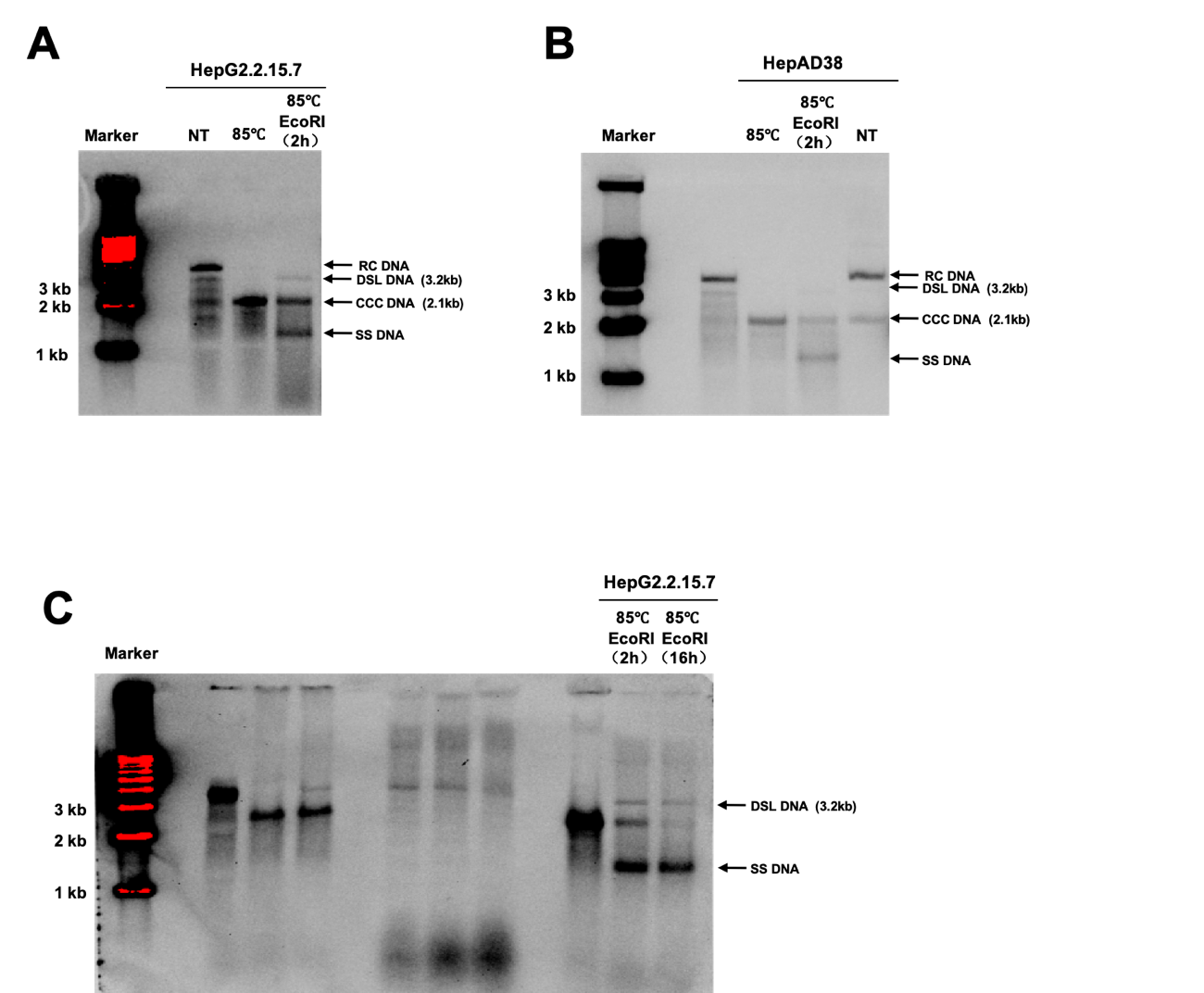
**

**Supplementary Fig. 1.** Original gel image with size marker. (**A**) HBV Hirt DNA was extracted from HepG2.2.15.7 (**A**) and HepAD38 (**B**) cells and detected by Southern blotting under the condition of no treatment, 85°C for 5 min, or 85°C for 5 min plus EcoRI 2 h digestion. (**C**) EcoRI digestion of HepG2.2.15.7 cells for 2 h and 16 h after 85°C treatment. A 1-kb one-step ladder (1–10 kbp) was used as marker. Lanes that are not described here were used for another experiment.

**
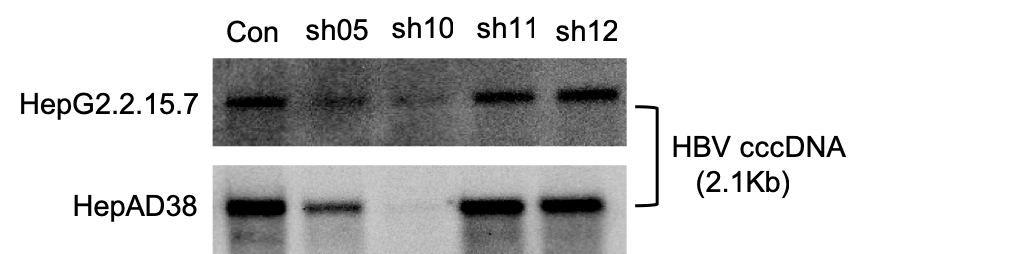
**

**Supplementary Fig. 2.** Southern blot analysis confirming decreased cccDNA levels in HepG2.2.15.7 and HepAD38 cells transfected with Jagged1 shRNA-05 and -10.


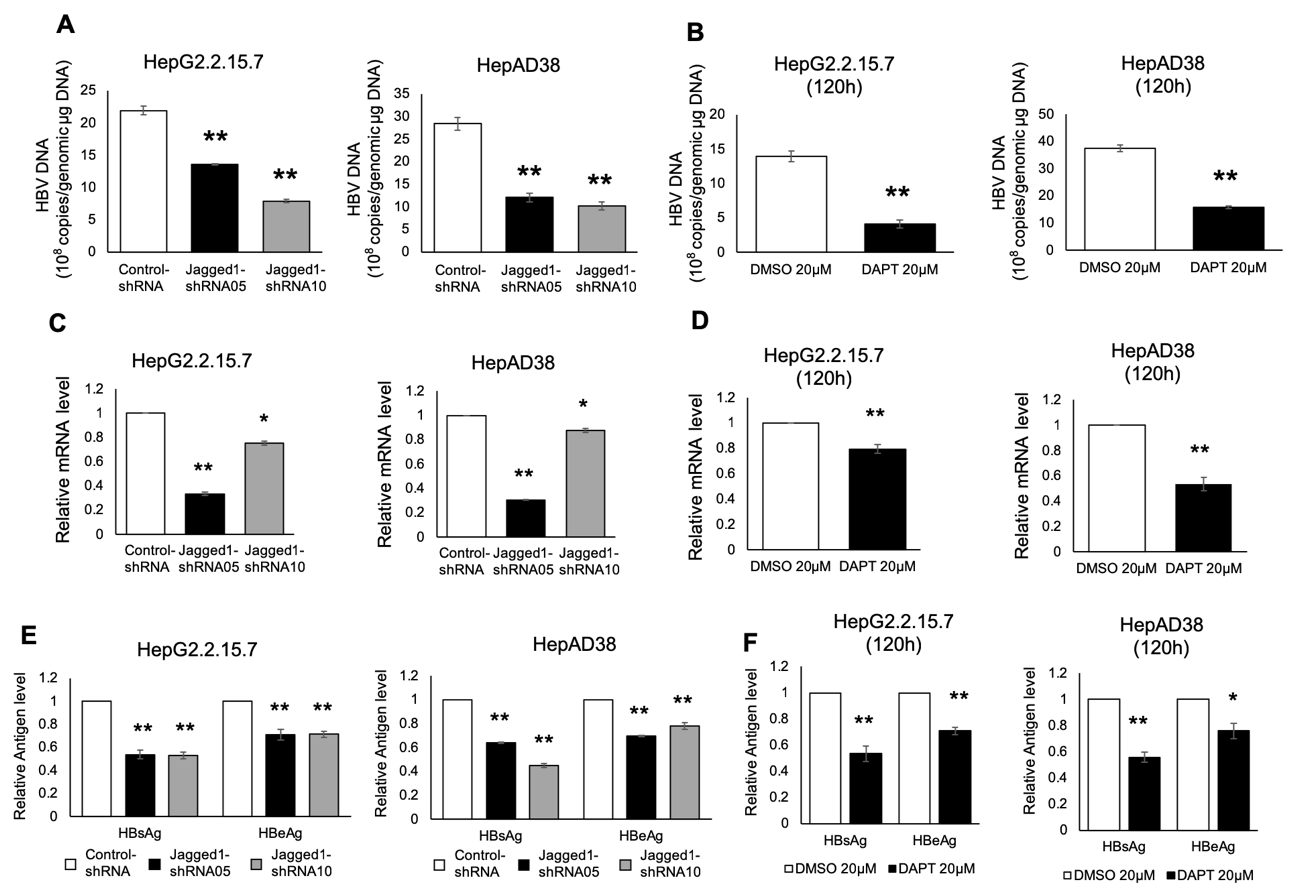


**Supplementary Fig. 3.** Notch inhibition markedly suppresses HBV replication. RTD-PCR quantification of the HBV DNA level after transfection of HepG2.2.15.7 and HepAD38 cells with Jagged1 shRNA (**A**) and treatment with 20 µM DAPT (**B**). Results are expressed as the number of DNA copies per μg (mean ± standard deviation) from three independent experiments. **P* < 0.05 and ***P* < 0.01 versus the corresponding control shRNA. RTD-PCR quantification of the HBV total RNA level after transfection of HepG2.2.15.7 and HepAD38 cells with Jagged1 shRNA (**C**) and treatment with 20 µM DAPT (**D**). Levels of hepatitis B surface antigen (HBsAg) and hepatitis B envelope antigen (HBeAg) after transfection with Jagged1 shRNA (**E**) and treatment with 20 µM DAPT (**F**) in HepG2.2.15.7 and HepAD38 cell supernatant. Quantitative gene expression data represent the mean ± standard deviation of three independent experiments and were normalized to the expression levels of 18sRNA. **P* < 0.05 and ***P* < 0.01 versus the corresponding control shRNA and DMSO (Control-shRNA, DMSO=1).


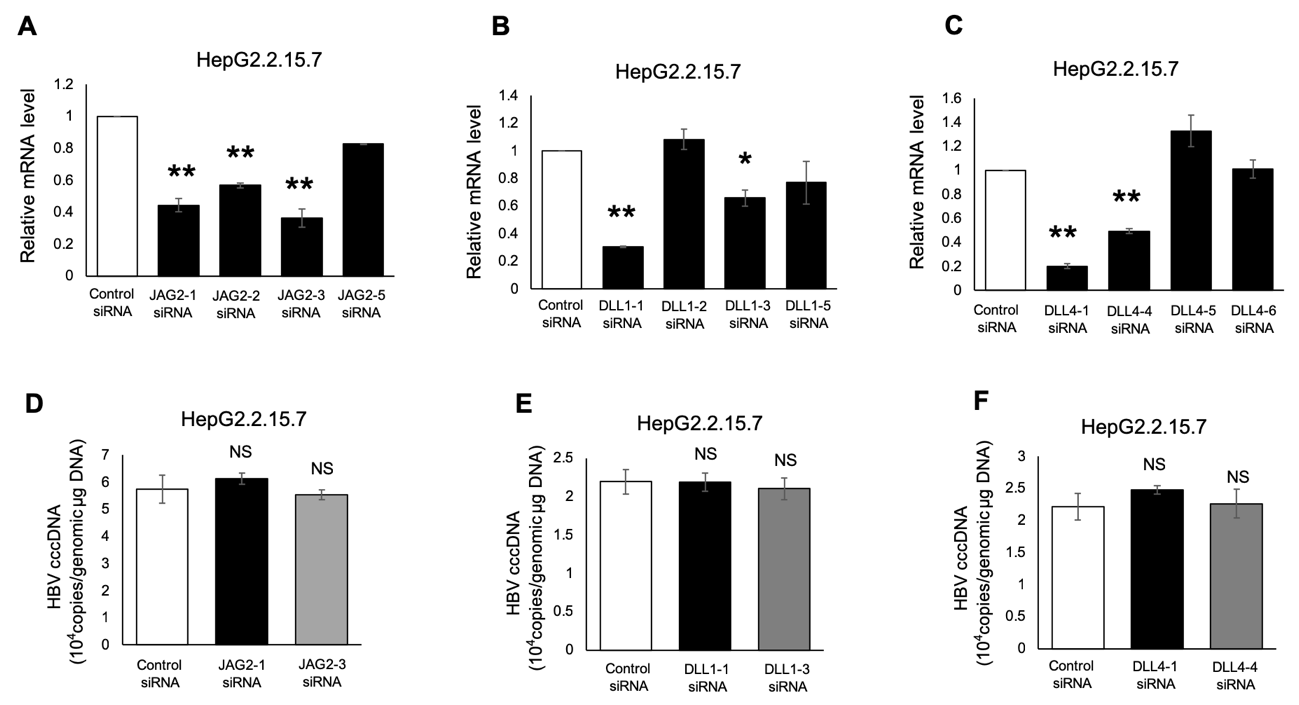


**Supplementary Fig. 4.** Inhibition of Notch ligands by specific siRNAs. RTD-PCR analysis of the gene expression levels of the Notch ligand JAG2 **(A)**, DLL1 **(B)**, and DLL4 **(C)** after transfection of HepG2.2.15.7 cells with the corresponding siRNAs. RTD-PCR quantification of cccDNA accumulation in HepG2.2.15.7 cells transfected with effective JAG2 **(D)**, DLL1 **(E)**, and DLL4 **(F)** siRNAs. cccDNA results are expressed as the number of cccDNA copies per μg (mean ± standard deviation) from three independent experiments. Quantitative gene expression data represent the mean ± standard deviation of three independent experiments and were normalized to the expression levels of human GAPDH. **P* < 0.05 and ***P* < 0.01 versus corresponding control siRNA values (Control-siRNA=1). Abbreviation: NS, not significant.


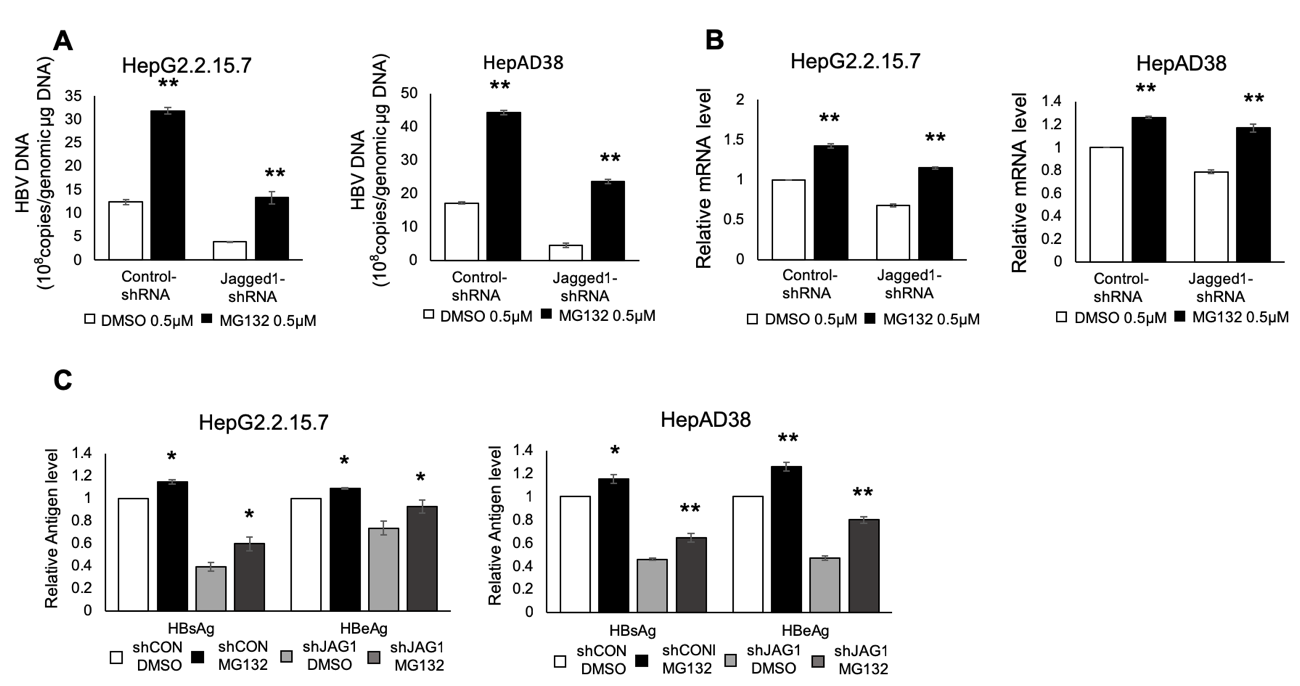


**Supplementary Fig. 5.** Proteasome inhibitor (MG-132) promotes HBV replication. (**A**) RTD-PCR quantification of the HBV DNA level after transfection of HepG2.2.15.7 and HepAD38 cells with Jagged1 shRNA with or without MG-132 (0.5 µM) treatment for 48 h. Results are expressed as the number of DNA copies per μg (mean ± standard deviation) from three independent experiments. **P* < 0.05 and ***P* < 0.01 versus the corresponding 0.5 µM DMSO. (**B**) RTD-PCR quantification of the HBV total RNA level after transfection of HepG2.2.15.7 and HepAD38 cells with Jagged1 shRNA with or without MG-132 (0.5 µM) treatment for 48 h. (**C**) HBsAg and HBeAg levels after transfection with Jagged1 shRNA with or without MG-132 (0.5 µM) treatment for 48 h in HepG2.2.15.7 and HepAD38 cell supernatant. Quantitative gene expression data represent the mean ± standard deviation of three independent experiments and were normalized to the expression levels of 18sRNA (HBV total RNA). **P* < 0.05 and ***P* < 0.01 versus the corresponding control shRNA and DMSO (Control-shRNA, DMSO=1). Abbreviations: shCON, Control-shRNA; shJAG1, Jagged1 shRNA.


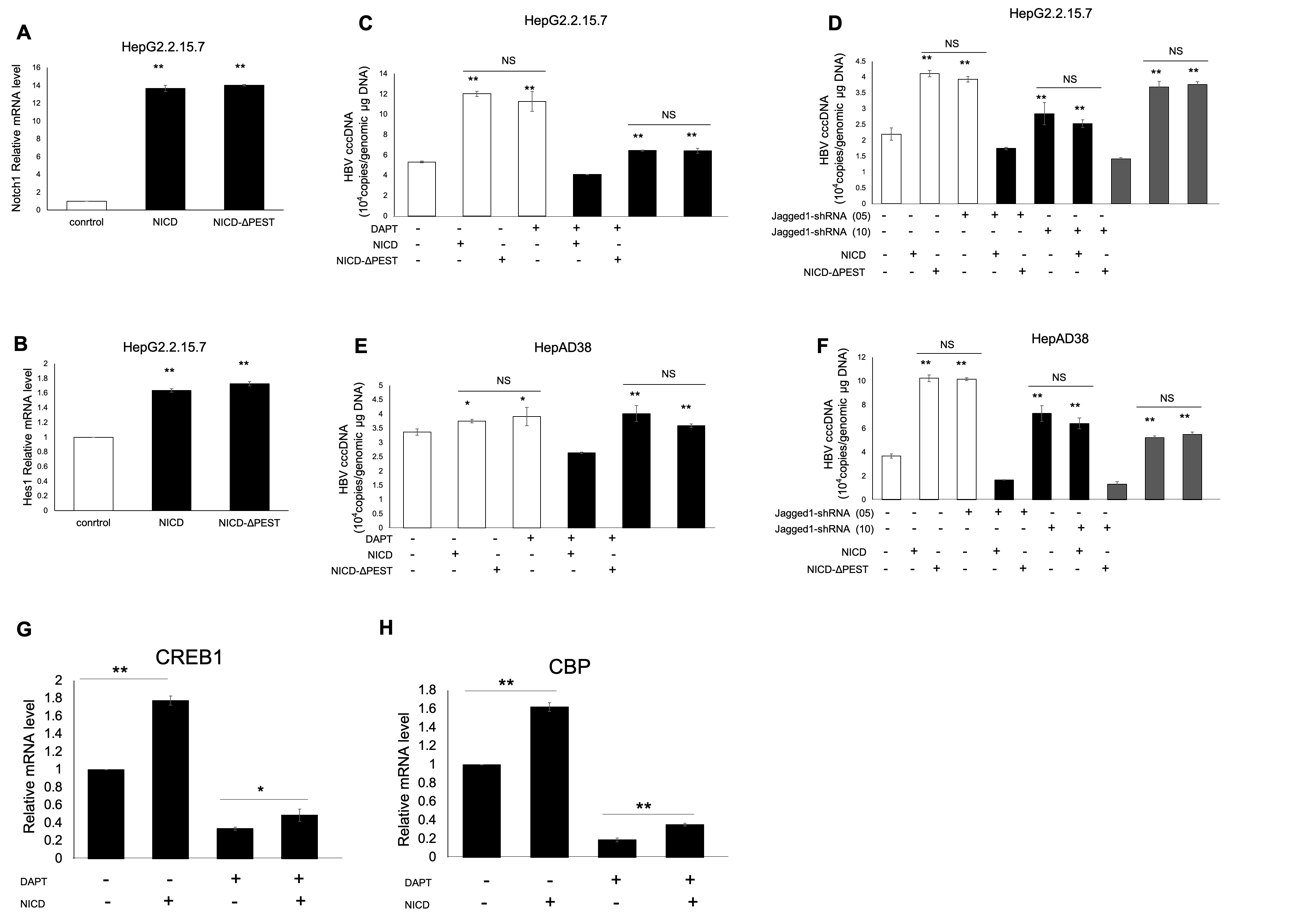


**Supplementary Fig. 6.** NICD overexpression facilitates HBV cccDNA. (**A**) RTD-PCR analysis of Notch1 (**A**) and Hes1 (**B**) gene expression levels after transfection of HepG2.2.15.7 cells with pCMV-XL4-NICD-HA and pCMV-XL4-NICD ΔPEST-HA plasmid. Quantitative gene expression data represent the mean ± standard deviation of three independent experiments and were normalized to the expression levels of GAPDH. **P* < 0.05 and ***P* < 0.01 versus the control pCMV-XL4 empty plasmid (Control=1). (**C**) RTD-PCR quantification of cccDNA accumulation in HepG2.2.15.7 cells transfected with pCMV-XL4-NICD-HA and pCMV-XL4-NICD ΔPEST-HA plasmid with or without DAPT treatment. (**D**) RTD-PCR quantification of cccDNA accumulation in HepG2.2.15.7 cells transfected with pCMV-XL4-NICD-HA and pCMV-XL4-NICD ΔPEST-HA plasmid with or without Jagged1-shRNA treatment. (**E**) RTD-PCR quantification of cccDNA accumulation in HepAD38 cells transfected with pCMV-XL4-NICD-HA and pCMV-XL4-NICD ΔPEST-HA plasmid with or without DAPT treatment. (**F**) RTD-PCR quantification of cccDNA accumulation in HepAD38 cells transfected with pCMV-XL4-NICD-HA and pCMV-XL4-NICD ΔPEST-HA plasmid with or without Jagged1-shRNA treatment. RTD-PCR quantification of the HBV total RNA level after transfection of HepG2.2.15.7 and HepAD38 cells with Jagged1 shRNA with or without MG-132 (0.5 µM) treatment for 48 h. Results are expressed as the number of DNA copies per μg (mean ± standard deviation) from three independent experiments. **P* < 0.05 and ***P* < 0.01 versus the control pCMV-XL4 empty plasmid. RTD-PCR analysis of CREB1 (**G**) and CBP (**H**) gene expression levels after transfection of HepG2.2.15.7 cells with pCMV-XL4-NICD-HA plasmid.

**
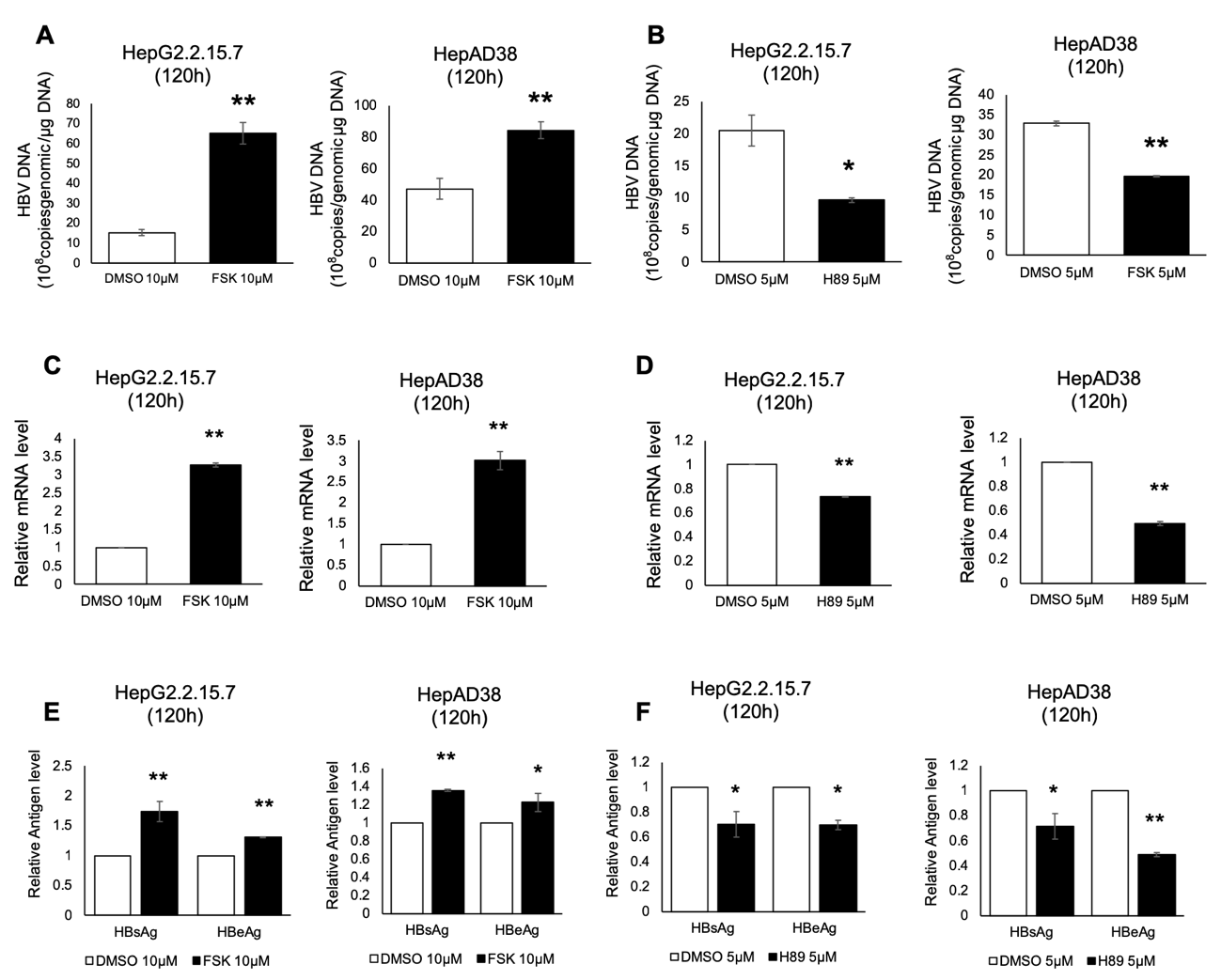
**

**Supplementary Fig. 7.** The CREB (pSer133CREB)-CBP cascade mediates HBV replication. RTD-PCR quantification of the HBV DNA level after treatment of HepG2.2.15.7 and HepAD38 cells with 10 µM Fsk (**A**) and 5 µM H-89 (**B**) for 120 h. Results are expressed as the number of DNA copies per μg (mean ± standard deviation) from three independent experiments. **P* < 0.05 and ***P* < 0.01 versus the corresponding DMSO. RTD-PCR quantification of the HBV total RNA level after treatment of HepG2.2.15.7 and HepAD38 cells with 10 µM Fsk (**C**) and 5 µM H-89 (**D**) for 120 h. HBsAg and HBeAg levels after 10 µM Fsk (**E**) and 5 µM H-89 (**F**) treatment for 120 h in HepG2.2.15.7 and HepAD38 cell supernatant. Quantitative gene expression data represent the mean ± standard deviation of three independent experiments and were normalized to the expression levels of 18sRNA. **P* < 0.05 and ***P* < 0.01 versus the corresponding DMSO (DMSO=1).


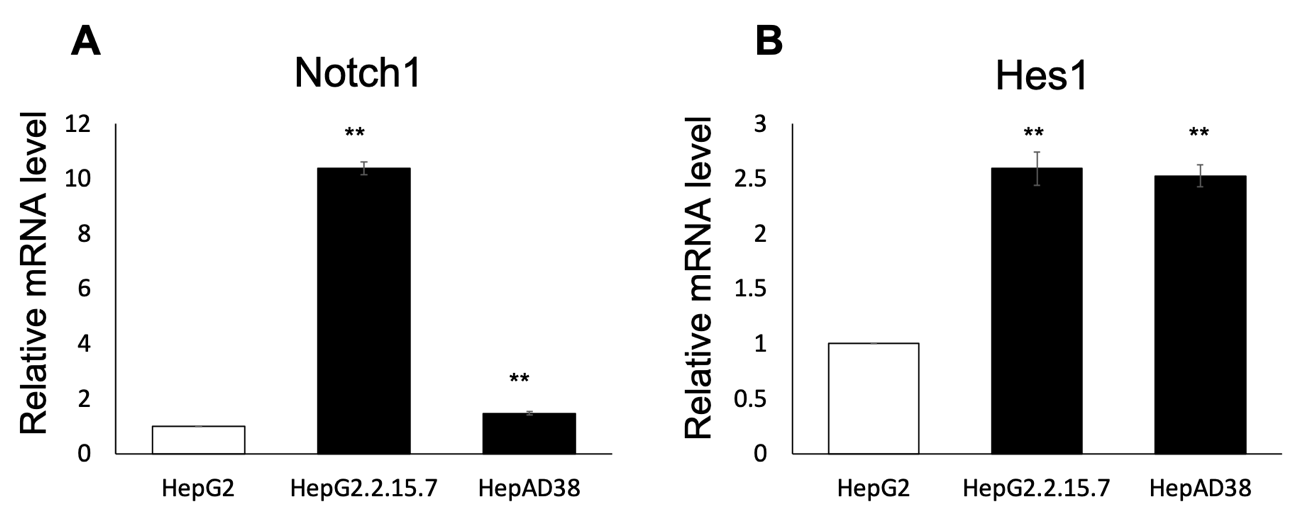


**Supplementary Fig. 8.** RTD-PCR analysis of Notch1 (**A**) and Hes1 (**B**) gene expression levels in HepG2, HepG2.2.15.7, and HepAD38 cells. Quantitative gene expression data represent the mean ± standard deviation of three independent experiments and were normalized to the expression levels of GAPDH. **P* < 0.05 and ***P* < 0.01 versus the level of HepG2 (HepG2 level=1).


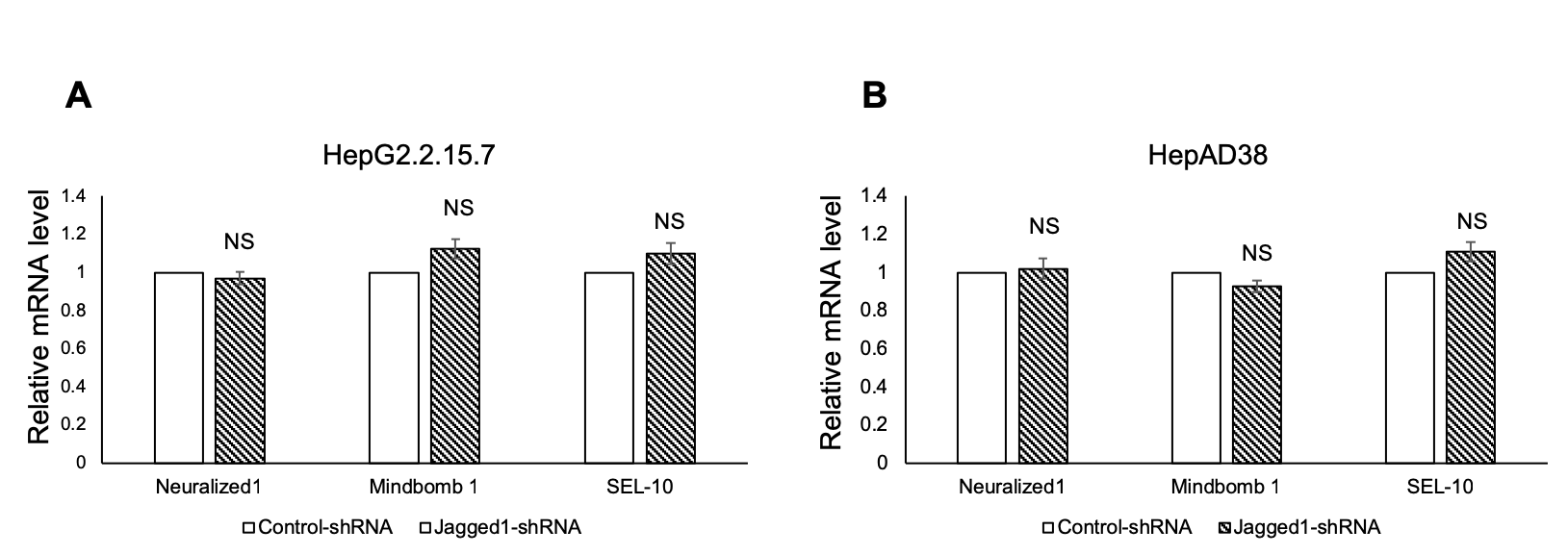


**Supplementary Fig. 9.** RTD-PCR analysis of gene expression after Jagged1 shRNA transfection in HepG2.2.15.7 (**A**) and HepAD38 (**B**) cells. Quantitative gene expression data represent the mean ± standard deviation of three independent experiments and were normalized to the expression levels of human GAPDH. **P* < 0.05 and ***P* < 0.01 versus the corresponding control shRNA values (control shRNA=1). Abbreviation: NS, not significant.


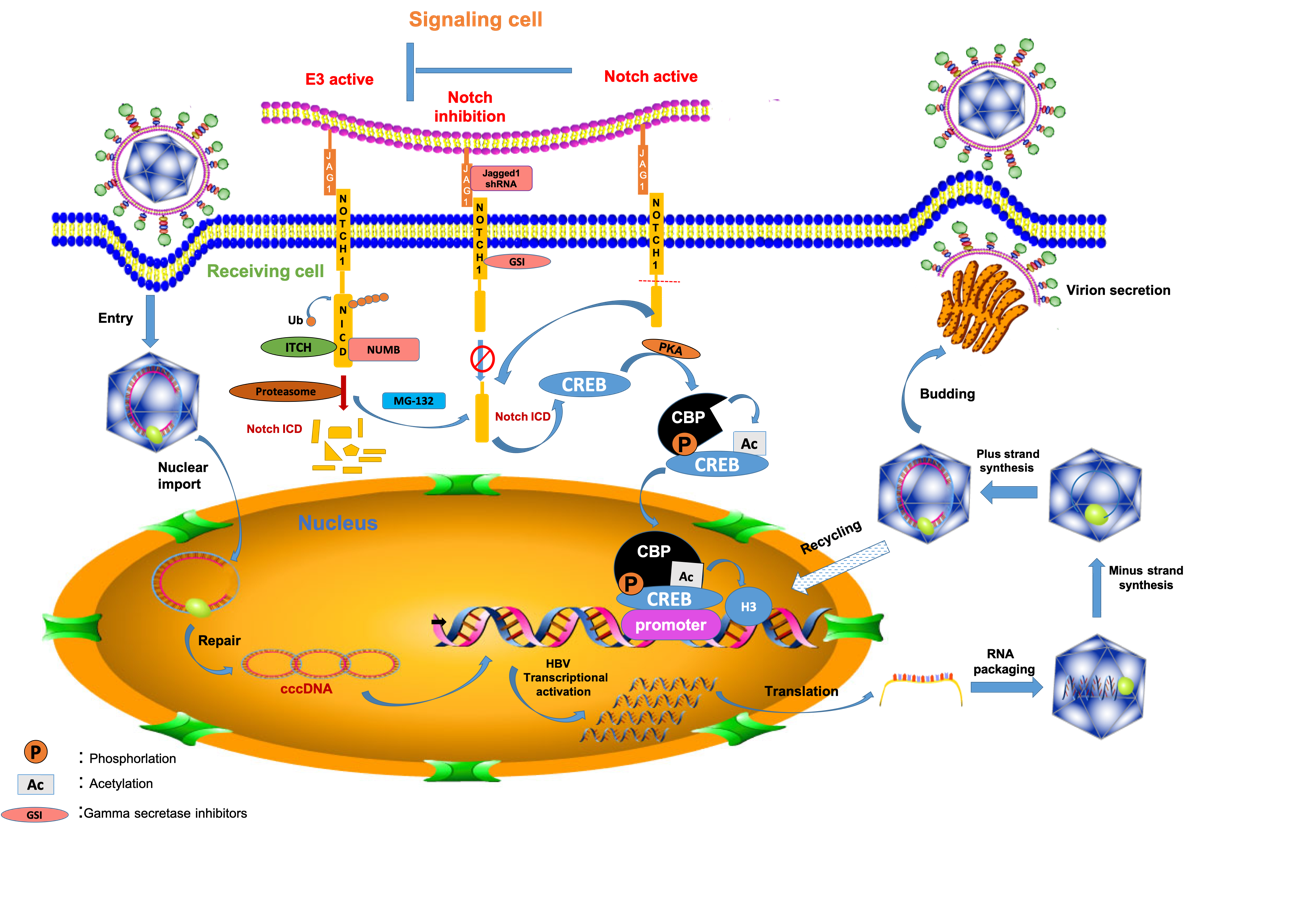


**Supplementary Fig. 10.** Model summarizing the proposed Jagged1-Notch1-CREB signaling axis modulation of HBV intrahepatic transcription and proteasomal degradation mediated by E3 ubiquitin ligases. Elements presumably connecting the Jagged1-Notch1-CREB-CBP circuit and Jagged1-Notch1-ITCH cascade according to this research and the existing literature are indicated by dotted lines.

**Supplementary Table 1**. Target sequences of the probes and primers used in this study.


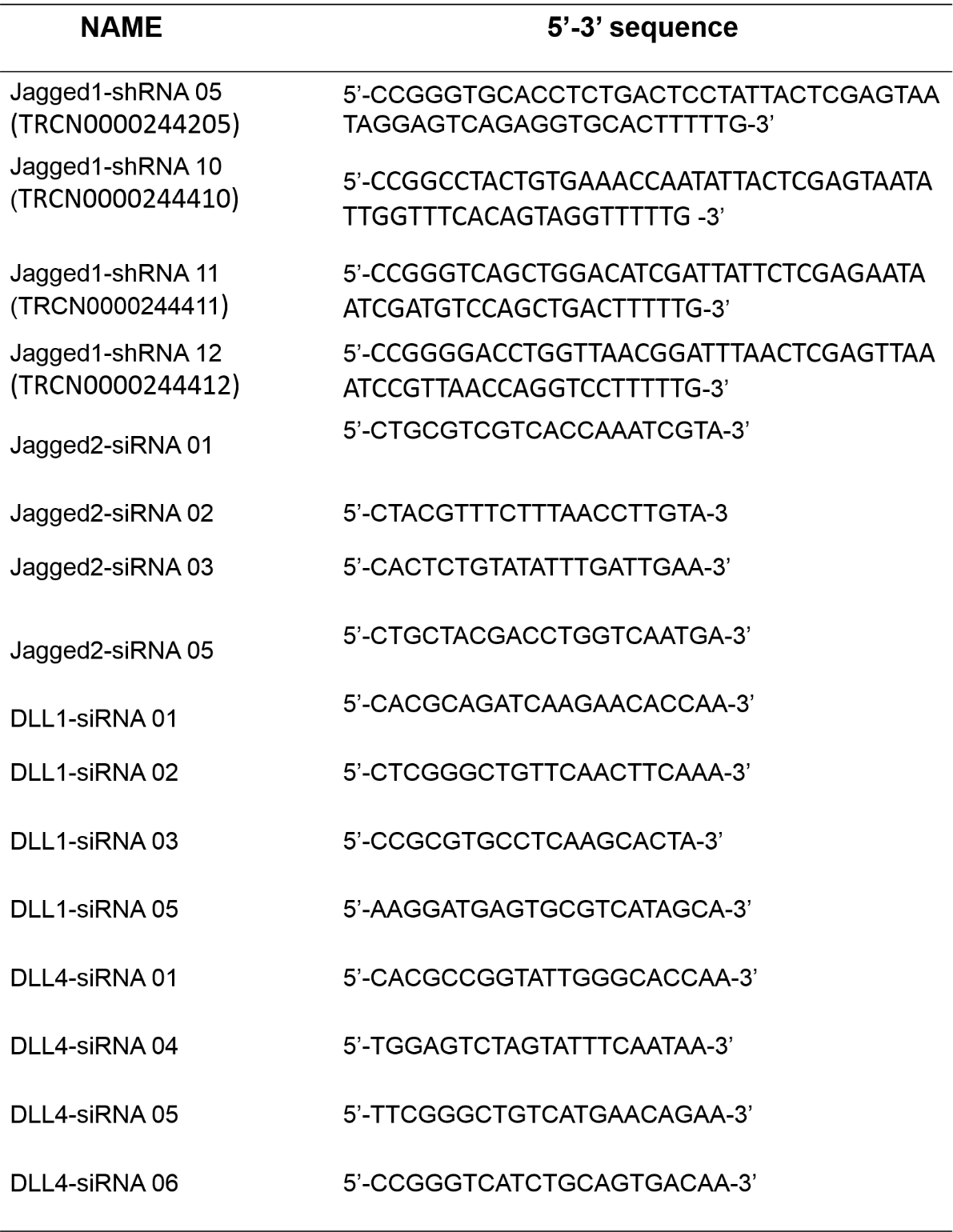


**Supplementary Materials and Methods**

**HBV DNA quantification**

HBV DNA was quantified with TaqMan Gene Expression Master Mix (Thermo Fisher Scientific, Waltham, MA) using a specific HBV DNA probe (5′-FAM-TATCGCTGGATGTGTCTGCGGCGT-TAMRA-3′), forward primer (5′-ACTCACCAACCTCCTCCTGTCCT-3′), and reverse primer (5′-GACAAACGGGCAACATACCT-3′) as follows: 50°C for 2 min, 95°C for 10 min, and 45 cycles of 95°C for 10 min and 65°C for 30 s. HBV DNA copy number was quantified relative to a known copy number of HBV DNA.

**HBV total RNA quantification**

For HBV total RNA, the cDNA samples were subjected to RTD-PCR using an RNA-specific probe (5’-FAM-CGTGTGCACTTCGCTTCACCTCTGC-MGB-3’) ^1^, forward primer (5’-GGGGCGCACCTCTCTTTACGCGGTC-3’), and reverse primer (5’-CAAGGCACAGCTTGGAGGCTTG-3’ (TaqMan); the relative pgRNA level for each sample was normalized to the expression of 18S rRNA.

**Enzyme-linked immunosorbent assay**

HBsAg and HBeAg levels in the culture medium were measured using enzyme-linked immunosorbent assay kits (CUSABIO, Houston, TX) according to the manufacturer’s instructions. All experiments were performed in triplicate.

**Transfection with siRNAs for Notch ligands**

GeneSolution siRNAs for Jagged2, DLL1, and DLL4 were used to silence gene expression (Qiagen, [Hilden](https://en.wikipedia.org/wiki/Hilden), [Germany](https://en.wikipedia.org/wiki/Germany)). The target sequences of these siRNAs are listed in Supplementary Table 1. For siRNA transfection, cells were seeded 1 day before transfection without antibiotics in culture medium and grown to 50% confluency on a 6-well plate. Transfection using Lipofectamine 2000 was performed following the manufacturer’s protocol. RNA interference efficiency was determined by RTD-PCR.

**NICD overexpression**

Notch 1 intracellular domain (NICD 1)-pcw107-V5 plasmid was purchased from Addgene (Watertown, MA). To generate the NICD-HA tag and ΔPEST-HA tag PCR fragment, PCR was performed using primers and NICD1-pcw107-V5 plasmid and PrimeSTAR GXL DNA polymerase (Takara, Shiga, Japan) following the manufacturer’s protocol. The sequence of the forward primer for NICD-HA and ΔPEST-HA was 5′-TAAGAATTCG CCGCCATGCG GCGGCAGCAT GGCCAGCTCT GGTTC-3′. The sequences of the reverse primer were 5′-AATAAGCTTT TAAGCGTAA TCTGGAACAT CGTATGGGTA CTTGAAGGCC TCCGGAATGC GGGCGAT-3′ for NICD-HA and 5′-AATAAGCTTT TAAGCGTAAT CTGGAACATC GTATGGGTAG TGGTTGAGGG GCACGGACGG AGACT-3′ for ΔPEST-HA. The amplified DNA fragment was then ligated into the EcoR1/HindIII cleavage pCMV-XL4 vector (Origene, Rockville, MD) using a Mighty Mix DNA Ligation Kit (Takara, Shiga, Japan). HBV-replicating cells were transfected with the appropriate amounts of pCMV-XL4-NICD-HA and pCMV-XL4-NICD ΔPEST-HA plasmids. RNA and hirt DNA were harvested at 48 h and 72 h after transfection. pCMV-XL4 empty plasmid was used as control.

1 Wang, J. *et al.* Serum hepatitis B virus RNA is encapsidated pregenome RNA that may be associated with persistence of viral infection and rebound. *J Hepatol* **65**, 700-710, doi:10.1016/j.jhep.2016.05.029 (2016).
